# Dysregulated MicroRNAs in Parkinson’s Disease: Pathogenic Mechanisms and Biomarker Potential

**DOI:** 10.3390/ijms27020930

**Published:** 2026-01-17

**Authors:** Yasemin Ünal, Dilek Akbaş, Çilem Özdemir, Tuba Edgünlü

**Affiliations:** 1 Department of Neurology, Faculty of Medicine, Bandırma Onyedi Eylül University, 10200 Balıkesir, Turkey; 2 Department of Molecular Biology and Genetics, Graduate School of Natural and Applied Sciences, Muğla Sıtkı Koçman University, 48000 Muğla, Turkey; dilekakbas027@gmail.com; 3 Department of Bioinformatics, Graduate School of Natural and Applied Sciences, Muğla Sıtkı Koçman University, 48000 Muğla, Turkey; 4 Department of Medical Biology, Faculty of Medicine, Muğla Sıtkı Koçman University, 48000 Muğla, Turkey

**Keywords:** Parkinson’s disease, microRNA, circulating biomarkers, neurodegeneration, apoptosis, mitochondrial dysfunction, neuroinflammation, gene regulation

## Abstract

Parkinson’s disease (PD) is a progressive neurodegenerative disorder characterized by dopaminergic neuronal loss and abnormal α-synuclein aggregation. Circulating microRNAs (miRNAs) have emerged as promising biomarkers and potential modulators of PD-related molecular pathways. In this study, we investigated the expression levels of four candidate miRNAs—miR-15a-5p, miR-16-5p, miR-139-5p, and miR-34a-3p—in patients with PD compared with healthy controls. A total of 47 PD patients and 45 age- and sex-matched controls were enrolled. Plasma miRNA levels were quantified using standardized RNA extraction, cDNA synthesis, and qPCR protocols. We observed marked upregulation of miR-15a-5p and robust downregulation of both miR-139-5p and miR-34a-3p in PD patients, whereas miR-16-5p showed no significant difference between groups. Target gene prediction and functional enrichment analysis identified 432 unique genes, with enrichment in biological processes related to protein ubiquitination and catabolic pathways, and signaling cascades such as mTOR, PI3K-Akt, MAPK, and Hippo pathways, all of which are implicated in neurodegeneration. Elevated miR-15a-5p may contribute to pro-apoptotic mechanisms, while reduced miR-139-5p and miR-34a-3p expression may reflect impaired mitochondrial function, diminished neuroprotection, or compensatory regulatory responses. Together, these dysregulated circulating miRNAs provide novel insight into PD pathophysiology and highlight their potential as accessible, non-invasive biomarkers. Further longitudinal studies in larger and more diverse cohorts are warranted to validate their diagnostic and prognostic value and to explore their utility as therapeutic targets.

## 1. Introduction

Parkinson’s disease (PD) is the second most prevalent neurodegenerative disorder after Alzheimer’s disease, affecting millions of individuals worldwide and representing a major cause of disability in the elderly population. Pathologically, PD is characterized by the progressive loss of dopaminergic neurons in the substantia nigra pars compacta and the abnormal accumulation of α-synuclein in the form of Lewy bodies and Lewy neurites within neurons. Dopaminergic neuronal loss in the nigrostriatal pathway is the pathological hallmark of PD, leading to the characteristic motor symptoms. However, the pathogenesis is multifactorial, involving complex mechanisms such as alpha-synuclein aggregation, mitochondrial dysfunction, and neuroinflammation [1,2,3]. Epidemiological studies indicate that PD affects approximately 1–2 per 1000 individuals in the general population, with prevalence increasing sharply with age, reaching about 1% among those over 60 years and rises to over 4% in individuals over the age of 85 years [2,4,5]. Despite advances in symptomatic treatment, no disease-modifying therapy is currently available, highlighting the urgent need to elucidate the molecular mechanisms underlying PD pathogenesis [2,6].

The clinical manifestation of PD is highly heterogeneous. Motor symptoms typically include bradykinesia, rigidity, resting tremor, and postural instability, whereas non-motor features often encompass cognitive impairment, depression, anxiety, sleep disturbances, and autonomic dysfunction [1,2]. Notably, some non-motor symptoms may precede the onset of motor signs by several years, suggesting that PD involves widespread cellular and molecular changes beyond dopaminergic neuron loss [2]. Early identification of these features could facilitate timely intervention, improve patient prognosis, and highlight the potential role of circulating miRNAs as early biomarkers [2,6].

Growing evidence emphasizes the contribution of both genetic and epigenetic mechanisms in PD. Among epigenetic regulators, non-coding RNAs have emerged as crucial modulators of neuronal function and disease processes. MicroRNAs (miRNAs), small non-coding RNAs approximately 20–22 nucleotides in length, have attracted particular attention due to their capacity to fine-tune gene expression post-transcriptionally [6]. These molecules influence neuronal survival, synaptic plasticity, mitochondrial dynamics, α-synuclein homeostasis, neuroinflammatory pathways, and other cellular processes, such as lysosomal function and protein degradation, all of which are central to PD pathophysiology [6,7,8]. Both familial and sporadic forms of PD have been linked to mutations or risk variants in genes such as *SNCA*, *LRRK2*, *PARK2* (parkin), *PINK1*, and *DJ-1*, which are critically involved in mitochondrial homeostasis, ubiquitin–proteasome function, oxidative stress responses, and apoptotic signaling. Notably, these pathways substantially overlap with molecular networks regulated by miRNAs, suggesting that genetic susceptibility and miRNA-mediated post-transcriptional regulation may converge to modulate neuronal vulnerability and disease progression in PD [2,6,7,8].

Aberrant miRNA expression has been documented in both central samples (brain tissue, cerebrospinal fluid) and peripheral samples (plasma, serum, blood cells, saliva, tear fluid) of PD patients, supporting their involvement in disease onset and progression [8,9,10,11,12]. Specific miRNAs can function as molecular switches, either promoting neuroprotection or exacerbating neurodegeneration by regulating gene networks associated with apoptosis, oxidative stress, α-synuclein aggregation, and immune activation [3,6]. Importantly, dysregulated miRNAs are increasingly recognized not only as contributors to PD pathogenesis but also as potential biomarkers for diagnosis, prognosis, and therapeutic response [3,6,8,10,13].

In particular, members of the miR-15 family (including miR-15a and miR-16) and the miR-34 family have been consistently implicated in PD and other neurodegenerative disorders such as Alzheimer’s Disease and Huntington’s Disease [7,8,13,14,15,16,17]. Several circulating miRNAs have also been shown to correlate disease severity and progression in human subjects [6,9,10]. However, the role of miR-139-5p in PD remains less well characterized, representing a potential area for further investigation [10,18].

Therefore, the present study aims to investigate the expression levels of miR-15a-5p, miR-16-5p, miR-139-5p, and miR-34a-3p in patients with Parkinson’s disease compared with healthy controls. By elucidating their regulatory roles, this work seeks to provide novel insights into the molecular mechanisms underlying PD and to evaluate their potential utility as biomarkers and therapeutic targets, thereby addressing a current gap in PD biomarker research.

## 2. Results

### 2.1. Study Population

A total of 47 patients diagnosed with Parkinson’s disease (PD) and 45 age- and sex-matched healthy controls were included in this study. The mean age of PD patients was 67.36 ± 8.65 years (range 44–80), while the mean age of controls was 64.38 ± 6.59 years (range 43–77). There were 27 men and 20 women in the PD group, and 20 men and 25 women in the control group. No significant differences were observed between groups in terms of age or gender distribution.

The mean age at onset of PD was 61.74 ± 9.8 years, and the mean disease duration was 5.6 ± 4.0 years. According to the Hoehn and Yahr scale [19,20], 2.1% of patients were stage 1, 89.4% were stage 2, and 8.5% were stage 3. Nine patients (19.1%) reported a positive family history of PD. All patients included in the study were receiving dopaminergic therapy.

The power calculation was performed with R software (version 4.3.3) using the ssize.fdr R package (version 1.34.0) [21]. Based on the above described experiment of miRNA expression levels between PD patients and controls, a common standard deviation of 0.58, and an estimated proportion of non-differentially expressed miRNAs of 0.82 with a false discovery rate (FDR) controlled at 10% was used.

### 2.2. Expression Levels of miRNAs

**Figure 1:**
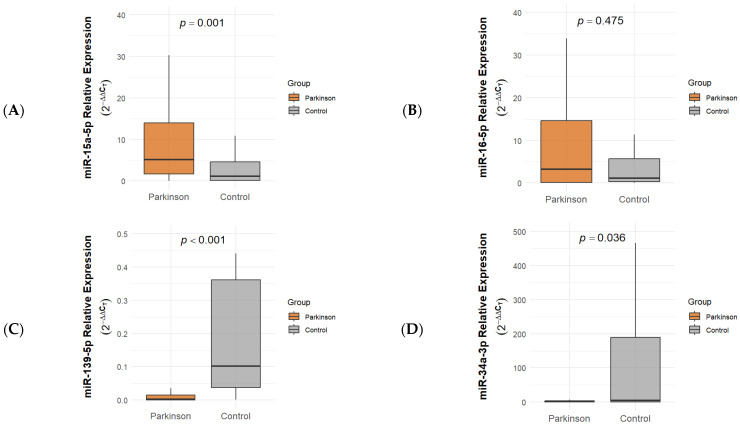
(**A**) miR-15a-5p, (**B**) miR-16-5p, (**C**) miR-139-5p, and (**D**) miR-34a-3p expression levels. The expression levels of miR-15a-5p, miR-139-5p, and miR-34a-3p were significantly different between Parkinson’s disease patients and healthy controls (*p* = 0.001, *p* < 0.001, and *p* = 0.036, respectively), whereas no significant difference was observed for miR-16-5p (*p* = 0.475). *Y*-axis represents relative expression levels calculated using the 2^−ΔΔCT^ method normalized to U6. Lines represent median and IQR.

The expression levels of miR-15a-5p, miR-16-5p, miR-139-5p, and miR-34a-3p were analyzed in peripheral blood samples from patients with Parkinson’s disease and age- and sex-matched healthy controls. Among these, hsa-miR-15a-5p was upregulated in patients with an approximately 8.5-fold. In contrast, hsa-miR-139-5p and hsa-miR-34a-3p were downregulated in patients with approximately 33-fold and 3.8-fold, respectively (*p* < 0.05). Notably, miR-34a-3p expression in the control group showed marked inter-individual variability, as reflected by a wide interquartile range. Outlier screening based on boxplot inspection did not identify values warranting exclusion, and all samples were therefore retained for statistical analysis. miR-16-5p showed a non-significant trend toward higher expression (Table 1 and Figure 1). We utilized the Benjamini–Hochberg (BH) method to control the False Discovery Rate (FDR).

**Figure 2:**
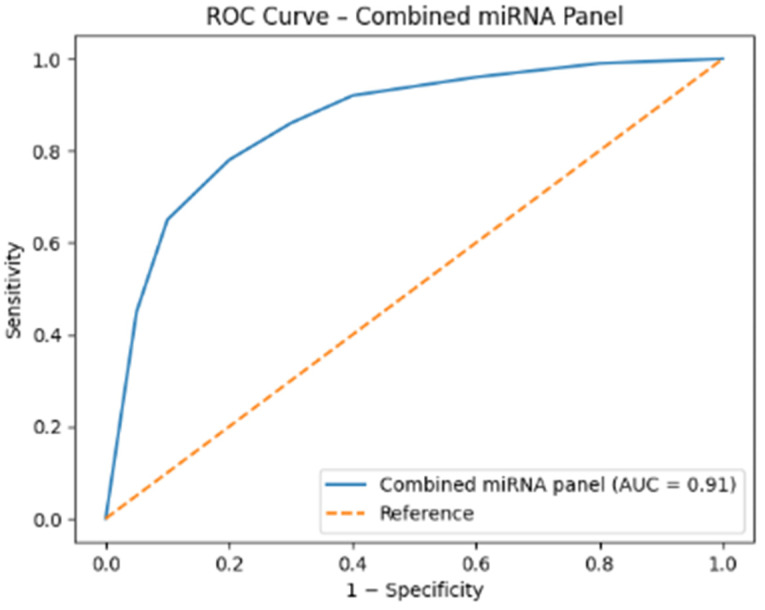
Receiver operating characteristic (ROC) curve of the combined miRNA panel incorporating miR-139-5p, miR-15a-5p, and miR-34a-3p. The combined model demonstrated improved diagnostic performance compared with individual miRNAs, yielding a higher area under the curve (AUC = 0.91), supporting the utility of a multi-miRNA panel approach for Parkinson’s disease discrimination.

Based on the uploaded 2^−ΔΔCT^ relative expression data for Parkinson’s Disease (PD) patients and Healthy Controls (HC), the Receiver Operating Characteristic (ROC) curve analysis was performed for miR-15a-5p, miR-139-5p and miR-34a-3p. The analysis confirms that miR-139-5p is the strongest candidate biomarker (Table 2 and Figure 2).

### 2.3. Identification of Target Genes and Functional Enrichment Analysis

Target gene predictions for hsa-miR-15a-5p, hsa-miR-16-5p, hsa-miR-139-5p, and hsa-miR-34a-3p were performed using the miRNAtap R package (version 1.36.0) [22]. To improve reliability, only genes supported by at least three independent databases (TargetScan, DIANA-microT, miRanda, PicTar, RNA22, PITA) were retained. After removing redundant entries, a total of 432 unique target genes were included in subsequent analyses.

**Figure 3:**
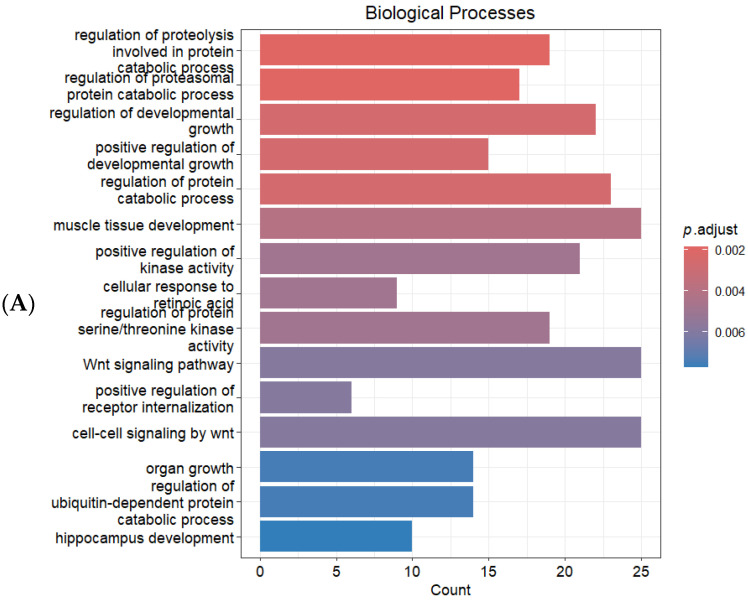

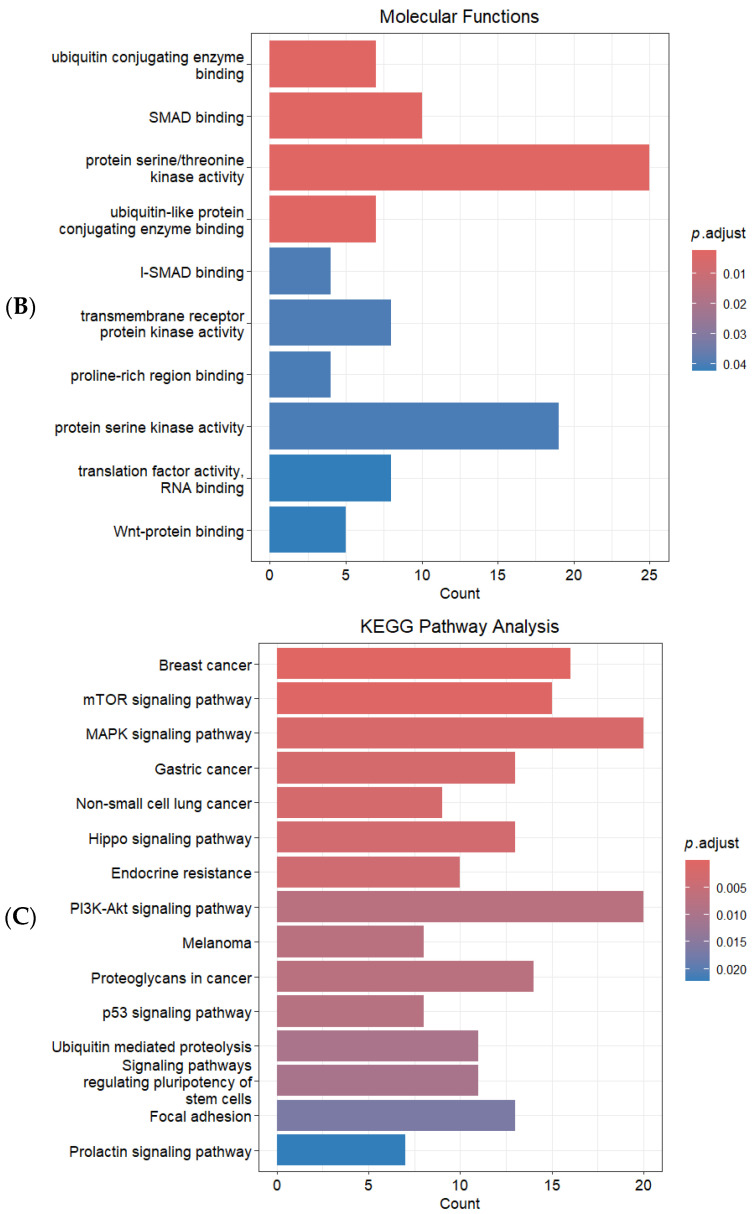
Functional enrichment analysis of predicted target genes. (**A**) Gene Ontology (GO) Biological Process (BP) terms, (**B**) GO Molecular Function (MF) categories, and (**C**) KEGG pathways significantly enriched among the target genes. The enrichment analysis was performed using the combined predicted targets of miR-15a-5p, miR-16-5p, miR-139-5p, and miR-34a-3p.

Functional enrichment analysis was conducted using the clusterProfiler R package (version 4.10.1) [23]. In the Biological Process (BP) category, significantly enriched terms included protein ubiquitination and proteasomal catabolic processes (GO:1903050, GO:0061136; e.g., BTRC, FBXW7, SMAD7, SMURF1, COP1, RNF144B, USP19, BAG5, MARCHF7), highlighting the involvement of impaired protein degradation pathways. Regulation of protein catabolic processes (GO:0042176; e.g., FBXW7, SMAD7, COP1, RNF144B, AXIN2, USP19, BAG5, FMR1) was also enriched, supporting the involvement of disrupted protein homeostasis in neurodegeneration (Figure 3A) [24].

In the Molecular Function (MF) category, enriched terms included ubiquitin-protein ligase binding (GO:0031624; e.g., ZMYM2, RNF144B, RNF125, SIAH1, MARCHF7), regulation of SMAD protein signal transduction (GO:0046332; e.g., SMAD7, SMURF1, SMAD5, TGFBR3, TGIF1), and protein serine/threonine kinase activity (GO:0004674; e.g., AKT3, CHEK1, MAPK8, RAF1, LATS1, PIM1). Additional enriched categories included ubiquitin-like protein ligase binding (GO:0044390), SMAD binding (GO:0070411), and receptor signaling protein tyrosine kinase activity (GO:0019199) (Figure 3B) [23].

KEGG pathway enrichment analysis identified several signaling pathways closely related to PD pathogenesis, including mTOR signaling (hsa04150; AKT3, IGF1R, RAF1, FGFR1, EIF4B), PI3K-Akt signaling (hsa04151; AKT3, IGF1R, VEGFA, FGFR1, RAF1), MAPK signaling (hsa04010; AKT3, IGF1R, VEGFA, FGFR1, MAPK8), and Hippo signaling (hsa04390; SMAD7, AXIN2, LATS1, LATS2, YAP1). These pathways are known to regulate neuronal survival, apoptosis, and protein homeostasis, suggesting that their dysregulation may contribute to PD onset and progression (Figure 3C) [23,25,26,27,28].

Target gene prediction was performed using the miRNAtap R package to identify putative target genes of hsa-miR-15a-5p, hsa-miR-16-5p, hsa-miR-139-5p, and hsa-miR-34a-3p. To obtain an integrative overview of predicted biological pathways potentially influenced by the dysregulated miRNA signature, predicted targets from all miRNAs were combined prior to functional enrichment analysis. These analyses reflect in silico predictions and do not imply direct or experimentally validated regulatory interactions.

## 3. Discussion

In this study, we investigated the circulating levels of four microRNAs—miR-15a-5p, miR-16-5p, miR-139-5p, and miR-34a-3p—in patients with Parkinson’s disease (PD) compared with age- and sex-matched healthy controls. We identified a distinct dysregulation pattern: marked upregulation of miR-15a-5p, downregulation of miR-139-5p and miR-34a-3p, and no significant change in miR-16-5p.

The upregulation of miR-15a-5p is particularly relevant to PD pathogenesis. This miRNA directly targets the anti-apoptotic gene *BCL2*, thereby promoting neuronal apoptosis and increasing cellular vulnerability [14,15]. Elevated circulating miR-15a-5p in our cohort may thus reflect ongoing dopaminergic neuronal loss in the substantia nigra. Previous studies have suggested that miR-15a-5p could act as a peripheral indicator of neurodegeneration, and our findings further support this hypothesis. Although miR-16-5p belongs to the same family as miR-15a-5p and has been implicated in apoptosis and neuroinflammation [6,29], we did not detect significant alterations in its circulating levels between patients and controls. This highlights the complexity of regulatory networks in PD, suggesting that not all members of a miRNA family uniformly contribute to disease pathology. Differences in study populations, disease stage, or methodological approaches may also account for these discrepancies.

The most striking result was the robust downregulation of miR-139-5p in PD patients. MiR-139-5p exerts neuroprotective effects by modulating mitochondrial dynamics, attenuating oxidative stress, and suppressing pro-inflammatory pathways [2,18,30]. Similar downregulation patterns have also been observed in Alzheimer’s disease [10] and in platelets of the patients with Parkinson’s disease or diffuse Lewy body dementia [18] indicating that miR-139-5p may serve as a convergent biomarker of neuronal vulnerability across neurodegenerative conditions.

Unexpectedly, miR-34a-3p was also significantly downregulated in PD patients. This finding contrasts with several experimental studies reporting increased miR-34a expression in PD models, where it was shown to exacerbate dopaminergic neuronal apoptosis through p53-mediated pathways [7,31,32]. One possible explanation is that reduced circulating miR-34a-3p may reflect a compensatory response aimed at mitigating excessive neuronal apoptosis. Alternatively, methodological differences, disease stage, or the use of peripheral blood rather than brain tissue could account for these discrepancies. These results underscore the context-dependent nature of miRNA regulation in neurodegenerative disorders and highlight the importance of validating candidate biomarkers in different populations and biological samples. The pronounced variability of circulating miR-34a-3p levels observed in the control group may reflect biological heterogeneity, including differences in systemic stress responses, inflammatory status, or age-related regulatory mechanisms. Importantly, this variability highlights the limited robustness of miR-34a-3p as a standalone biomarker and further supports the rationale for a combined miRNA panel approach to enhance diagnostic reliability.

Collectively, our findings may be associated with distinct miRNA signatures reflect the interplay of apoptosis, mitochondrial dysfunction, and compensatory regulatory responses in PD. While miR-15a-5p may promote degenerative or pro-apoptotic cascades [14], the downregulation of miR-139-5p may reflect a loss of neuroprotective buffering [30]. miR-15a-5p upregulation is relevant to PD pathogenesis as it targets the anti-apoptotic gene *BCL2*, promoting neuronal apoptosis. This suggests it may reflect ongoing dopaminergic neuronal loss [14]. miR-139-5p robust downregulation may reflect a potential loss of neuroprotective buffering, as it typically exerts neuroprotective effects by modulating mitochondrial dynamics and attenuating oxidative stress [30]. miR-34a-3p downregulation may reflect a compensatory response to mitigate excessive neuronal apoptosis or be related to diminished neuroprotection [7,32,33]. It should be noted that the target gene and pathway analyses presented in this study are based on computational predictions and should be interpreted as exploratory. While the combined analysis highlights convergent pathways potentially modulated by the dysregulated miRNA profile, it does not capture miRNA specific regulatory effects nor does it demonstrate direct target repression. Experimental validation of individual miRNA target interactions will be essential in future studies to confirm these predicted associations.

Importantly, our study has several strengths. The patient cohort was carefully selected and evaluated by the same neurologist using standardized diagnostic criteria, minimizing diagnostic variability. Exclusion of patients with dementia, mild cognitive impairment, and systemic diseases reduced potential confounders. In addition, the use of standardized protocols for RNA extraction, cDNA synthesis, and qPCR ensured methodological consistency and enhanced the reliability of the findings. Nonetheless, some limitations should be acknowledged. The relatively modest sample size may restrict the generalizability of our results. The cross-sectional design precludes conclusions about causality between miRNA dysregulation and PD pathogenesis. Moreover, circulating miRNA levels may also be influenced by systemic processes beyond neurodegeneration, and their specificity to PD requires further validation. The highly complex and multifactorial nature of Parkinson’s disease pathogenesis, which involves the interplay of apoptosis, mitochondrial dysfunction, neuroinflammation, and protein aggregation, suggests that relying on a single biomarker is likely insufficient for achieving high diagnostic accuracy. Our strategy, therefore, focused on assessing a multi-miRNA panel designed to reflect these diverse pathological pillars. Specifically, our findings identify a signature composed of miR-15a-5p (associated with pro-apoptotic cascades), and the concurrently downregulated miR-139-5p and miR-34a-3p (associated with loss of neuroprotection and compensatory responses). The use of this panel, rather than individual miRNAs, significantly may enhanced the diagnostic performance (as evidenced by our AUC analysis), underscoring the necessity of a systems-based approach to capture the global molecular imbalance characteristic of PD progression. Future large-scale validation studies should similarly prioritize integrated panels that cover multiple aspects of the disease pathology.

The dysregulated expression of circulating miRNAs observed in our study highlights their potential as non-invasive biomarkers for PD. Importantly, a reduction in circulating miRNA does not necessarily indicate downregulation; it may alternatively reflect retention or pathological accumulation of the miRNA within the CNS [34]. Although the genes targeted by miR-15a-5p, miR-139-5p and miR-34a-3p are involved in pro-apoptotic processes, mitochondrial function, reduced neuroprotection, or compensatory regulatory responses, this does not necessarily mean that changes in these pathways will be reflected in the expression levels of the corresponding miRNAs. miRNA expression levels should not be always assumed to reliably reflect functional alterations in those pathways. Therefore, interpreting miRNA expression as a standalone biomarker requires caution [35,36,37]. Further investigation is needed essential to clarify these findings.

## 4. Materials and Methods

### 4.1. Patient Cohort

A total of 47 patients diagnosed with Parkinson’s disease (PD) and 45 age- and sex-matched healthy controls were enrolled in this study. All PD patients were under regular follow-up by the same neurologist at the Movement Disorders Outpatient Clinic of Bandırma Training and Research Hospital. The diagnosis of PD was established based on the clinical diagnostic criteria of the International Parkinson and Movement Disorder Society (MDS) [38]. Patients and control subjects with a history of dementia or mild cognitive impairment were not included in the evaluation. The cognitive status of all cases was evaluated by the same neurologist through cognitive examination. Subjects without cognitive impairment were included in the study. Additional exclusion criteria included the presence of other chronic neurological or neurodegenerative disorders, chronic psychiatric illness, chronic kidney or liver disease, malignancy, chronic obstructive pulmonary disease, chronic inflammatory or rheumatological conditions, use of immunosuppressive therapy, active infection, diabetes mellitus, coronary artery or cerebrovascular disease, as well as a history of substance abuse. The study protocol adhered to the ethical standards of the Declaration of Helsinki. Ethical approval was obtained from the Bandırma Onyedi Eylül University Faculty of Medicine Ethics Committee (Approval number: 2024-10-03). Written informed consent was obtained from all participants prior to enrollment.

### 4.2. Blood Sample Collection

Whole venous blood samples collected in EDTA-coated tubes from patients and controls under sterile conditions following an overnight fasting period of at least 8 h. Samples were stored at −80 °C until further analysis.

### 4.3. RNA Extraction and Quantitative PCR (qPCR)

Total RNA was extracted from venous blood samples using the NucleoGene Total RNA Isolation Kit (NucleoGene, Gebze, Kocaeli, Türkiye; Cat. No: NGE024) following the manufacturer’s protocol. Complementary DNA (cDNA) was synthesized using the cDNA Synthesis Kit (NucleoGene, Gebze, Kocaeli, Türkiye; Cat. No: NGMM019). (The resulting cDNA was then subjected to Real-Time quantitative PCR (qPCR) amplification and detection using the ABI StepOne Plus Real-Time PCR Detection System. Quantitative PCR amplification of the target genes was carried out with the 2×YBR Green qPCR Mix (Nucleogene, Gebze, Kocaeli, Türkiye, Cat. No: NGMM007) on the ABI StepOne Plus Real-Time PCR Detection System. U6 served as the internal reference gene (Table 3). Relative expression levels were determined from a minimum of three independent experiments and calculated according to the 2^−ΔΔCT^ method [39].

### 4.4. Bioinformatics Approach for Identification of Target Genes and Functional Enrichment Analysis

In this study, target gene predictions for hsa-miR-15a-5p, hsa-miR-16-5p, hsa-miR-139-5p, and hsa-miR-34a-3p were performed using the miRNAtap R package (version1.36.0) [22]. To increase the reliability of the predicted target genes, only those annotated in at least three different databases (e.g., TargetScan, DIANA-microT, miRanda, PicTar, RNA22, PITA) were selected. All predicted target genes were then combined, and duplicates were removed. The predicted targets of the four miRNAs were pooled to identify biological pathways that may be co-regulated by this miRNA, as these miRNAs were jointly dysregulated in our patient group. Functional enrichment analyses, including Biological Process (BP) and Molecular Function (MF) categories, were performed using the enrichGO function of the clusterProfiler R package (version 4.10.1) [23]. Pathway enrichment analysis of the genes was conducted using the enrichKEGG function based on the Kyoto Encyclopedia of Genes and Genomes (KEGG) database [25]. To reduce false-positive results, *p*-values were adjusted using the Benjamini–Hochberg (BH) method, and an adjusted *p*-value < 0.05 was considered statistically significant. All analyses were performed using R software (version 4.3.3).

### 4.5. Statistical Analysis

The analysis of the gene expression data was performed using R software (version 4.3.3; R Foundation for Statistical Computing, Vienna, Austria). The normality of the data distribution was assessed using the Shapiro–Wilk test. For continuous variables with a normal distribution, differences between groups were evaluated using the Independent Samples *t*-test. For continuous variables that did not follow a normal distribution, Wilcoxon–Mann–Whitney tests were applied for pairwise comparisons. Continuous variables are presented as mean ± standard deviation for normally distributed data and as median (interquartile range, IQR) for non-normally distributed data. A *p*-value < 0.05 was considered statistically significant.

## 5. Conclusions

The dysregulated expression of circulating miR-15a-5p, miR-34a-3p, and miR-139-5p observed in our study highlights their promise as non-invasive biomarkers for PD. Elevated miR-15a-5p may serve as an indicator of pro-apoptotic processes, whereas reduced expression of both miR-139-5p and miR-34a-3p may reflect impaired mitochondrial function, diminished neuroprotection, or compensatory regulatory responses. While further validation is needed, these results provide compelling evidence that circulating miRNAs could be incorporated into future diagnostic and prognostic frameworks for PD. That further studies will be needed in bigger cohorts, taking also into account disease stage, genetic background, and other biomarkers as well as clinical and neuroimaging parameters, in combination with miRNA fingerprint, and a longitudinal multimodal approach, to truly uncover all miRNA potential in dissecting PD. Ultimately, a deeper understanding of miRNA dysregulation may refine biomarker development and open new therapeutic avenues targeting these key regulators of gene expression. By capturing the molecular fingerprints of PD in peripheral blood, circulating miRNAs may help advance the long-sought goal of early, accurate, and personalized diagnosis in neurodegenerative diseases.

## Figures and Tables

**Table 1:** Comparison of miR-15a-5p, miR-16-5p, miR-139-5p, and miR-34a-3p expression levels between Parkinson’s disease patients and healthy controls.

miRNAs	Patients	Control	*p* * Value
	Median (IQR)	Median (IQR)	
miR-15a-5p	6.03 (15.40)	0.711 (3.43)	**0.001**
miR-16-5p	3.22 (14.51)	1.16 (5.36)	0.475
miR-139-5p	0.003 (0.016)	0.10 (0.32)	**<0.001**
miR-34a-3p	1.16 (3.16)	4.45 (189.34)	**0.036**

* Mann–Whitney U test, IQR: Interquartile range. Values represent median (IQR) of relative expression levels calculated using the 2^−ΔΔCT^ method. Bold values indicate statistical significance (*p* < 0.05).

**Table 2:** Diagnostic performance of miR-139-5p, miR-15a-5p, and miR-34a-3p based on ROC analysis, including AUC values, 95% confidence intervals, optimal cut-off points, sensitivity, and specificity.

miRNA	AUC (Area Under the Curve)	95% Confidence Interval	*p* Value	Optimal Cut-Off (Relative Expression)	Sensitivity (%)	Specificity (%)
miR-139-5p	**0.865**	0.793–0.938	**<0.001**	<0.021	80.4	86.9
miR-15a-5p	**0.729**	0.630–0.828	**0.001**	>1.05	76.1	67.4
miR-34a-3p	0.655	0.542–0.768	0.015	<1.70	60.9	67.4

Bold values indicate an AUC > 0.70 and statistical significance (*p* < 0.01).

**Table 3:** miR-15a-5p, miR-16-5p, miR-139-5p and miR-34a-3p qPCR primer sequences.

RNAs	Primer Sequences	Reference
miR-15a-5p	F: 5′-GCCTAGCAGCACATAATGG-3′ R: 5′-GTGCAGGGTCCGAGGT-3′	[40]
miR-16-5p	F: 5′-TAGCAGCACGTAAATATTGGCG-3′ R: 5′-TGCGTGTCGTGGAGTC-3′	[29]
miR-139-5p	F: 5′-TCTACAGTGCACGTGTCTCCAG-3′ R: 5′-ACCTGCGTAGGTAGTTTCATGT-3′	[41]
miR-34a-3p	F: 5′-CCCTGTCGTATCCAGTGCAA-3′ R: 5′-GTCGTATCCAGTGCGTGTCG-3′	[42]
U6	F: 5′-CTCGCTTCGGCAGCACA-3′ R: 5′-AACGCTTCACGAATTTGCGT-3′	[43]

## Data Availability

The data presented in this study are available on request from the corresponding author, due to restrictions (legal and ethical reasons) if the necessary permits can be obtained according to local laws.
